# Highights in the History of Epilepsy: The Last 200 Years

**DOI:** 10.1155/2014/582039

**Published:** 2014-08-24

**Authors:** Emmanouil Magiorkinis, Aristidis Diamantis, Kalliopi Sidiropoulou, Christos Panteliadis

**Affiliations:** ^1^Office for the Study of Hellenic Naval Medicine, Naval Hospital of Athens, Deinokratous 70, 11527 Athens, Greece; ^2^Division of Paediatric Neurology and Developmental Medicine, Aristotle University of Thessaloniki, AHEPA Hospital, Stilp Kiriakidi 1, 54634 Thessaloniki, Greece

## Abstract

The purpose of this study was to present the evolution of views on epilepsy as a disease and symptom during the 19th and the 20th century. A thorough study of texts, medical books, and reports along with a review of the available literature in PubMed was undertaken. The 19th century is marked by the works of the French medical school and of John Hughlings Jackson who set the research on epilepsy on a solid scientific basis. During the 20th century, the invention of EEG, the advance in neurosurgery, the discovery of antiepileptic drugs, and the delineation of underlying pathophysiological mechanisms, were the most significant advances in the field of research in epilepsy. Among the most prestigious physicians connected with epilepsy one can pinpoint the work of Henry Gastaut, Wilder Penfield, and Herbert Jasper. The most recent advances in the field of epilepsy include the development of advanced imaging techniques, the development of microsurgery, and the research on the connection between genetic factors and epileptic seizures.

## 1. Introduction 

The history of epilepsy is intermingled with the history of human existence; the first reports on epilepsy can be traced back to the Assyrian texts, almost 2,000 B.C. [1]. Multiple references to epilepsy can be found in the ancient texts of all civilizations, most importantly in the ancient Greek medical texts of the Hippocratic collection. For example, Hippocrates in his book* On Sacred Disease* described the first neurosurgery procedure referring that craniotomy should be performed at the opposite side of the brain of the seizures, in order to spare patients from “phlegma” that caused the disease [2]. However, it was not until the 18th and 19th century, when medicine made important advances and research on epilepsy was emancipated from religious superstitions such as the fact that epilepsy was a divine punishment or possession [3, 4]. At the beginning of the 18th century, the view that epilepsy was an idiopathic disease deriving from brain and other inner organs prevailed. One should mention the important work in this field by William Culen (1710–1790) and Samuel A. Tissot whose work set the base of modern epileptology describing accurately various types of epilepsies.

## 2. Anatomy and Physiology of Epilepsy

### 2.1. Evolution of Thoughts around the Pathophysiology and Causes of Epilepsy

At the beginning of the 19th century, physicians from the French medical school started to publish their research in the field of epileptology; famous French physicians published their works on epilepsy such as Maisonneuve (1745–1826) [5], Calmeil (1798–1895) [6], and Jean-Etienne Dominique Esquirol (1772–1840). Maisonneuve stressed the importance for hospitalization of epileptic patients, categorized epilepsy into idiopathic and sympathetic and described the so-called sensitive aura of sympathetic epilepsy. Esquirol distinguished between petit and grand mal and along with his pupils Bouchet and Cazauvieilh studied systematically insanity and epilepsy conducting clinical and postmortem studies [3].

**Figure 1:**
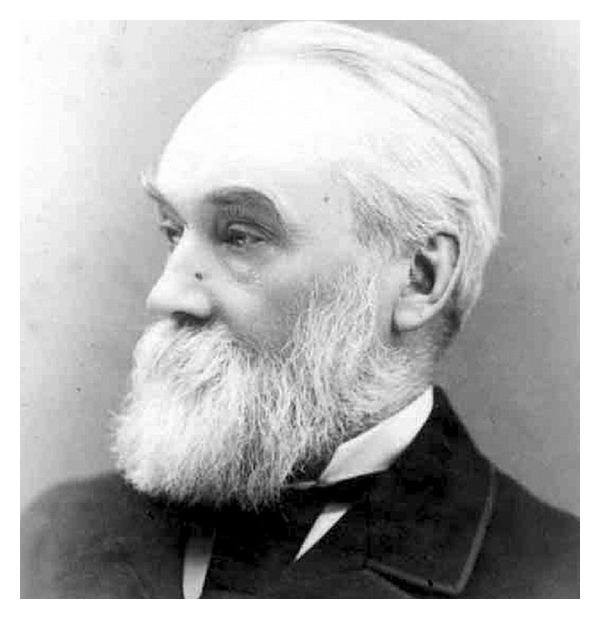
John Hughlings Jackson (1835–1911) (adopted by public domain at http://www.denstoredanske.dk/).

During the second half of the 19th century, medicine focused on the delineation of pathophysiology of epilepsy and the topographic localization of epileptic seizures. Important works on epileptogenesis, aetiology, and taxonomy of epilepsy were published by prestigious physicians such as Théodore Herpin (1799–1865) in 1852 and 1867, Louis Jean François Delasiauve (1804–1893) in 1854, John Russell Reynolds (1828–1896) in 1861, and in 1881 by Sir William Richard Gowers (1845–1915). Regarding the delineation of the epileptic mechanisms, the proof that epilepsy derives from the brain came from the work of physiologist Fritsch (1838–1927) and psychiatrist Hitzig (1838–1907); in their paper entitled “*On the Electric Excitability of the Cerebrum”* they presented experiments in which they provoked seizures by electric stimulation in the brain cortex of dogs [7]. The work, however, of John Hughling Jackson (1835–1911) (Figure 1), set the scientific base of epileptology [3]. Jackson studied epilepsy on pathological and anatomical basis. His* Study of Convulsions *was the culmination of his research stressing the existence of localised lesions on cortex involved in epileptic convulsions. In 1873, Jackson gave the following definition for epilepsy: “Epilepsy is the name for occasional, sudden, excessive, rapid and local discharges of grey matter” [3].

Epileptology, based on the work of Jackson and other eminent doctors of the 19th century, such as John Simon (1816–1904), John Russell Reynolds (1828–1896), Samuel Wilks, William Richard Gowers (1845–1915), Adolf Kussmaul (1822–1902), and Adolf Tenner, expanded and made important steps towards the elucidation of the pathophysiology of the disease and in the field of therapeutics [3].

At the beginning of the 20th century, Santiago Ramón y Cajal (1852–1934), a Spanish pathologist, histologist, and neuroscientist, made important advances in the field of the microscopic structure of the brain and the nervous system. He was the first to describe the structure of neurons and synapses, a hallmark finding in the history of neurology. His findings were the culmination of efforts which began in 1887, when he started employing the Golgi staining in the study of the nervous system. As a reward of his efforts, Cajal, in 1906, received the Nobel Prize [8].

In 1907, Gowers published his famous book* The Borderlands of Epilepsy* [9] focusing on faints, vagal and vasovagal attacks, migraine, vertigo, and some sleep symptoms, especially narcolepsy. In 1914, Dale (1875–1968) identified acetylcholine [10], the first neurotransmitter, a discovery confirmed later in 1921 by Loewi (1873–1961) who initially named it* Vagusstoff*, since it was released by the vagus nerve [11–13].

During the 1920s, Lennox (1884–1960) and Cobb (1887–1968) focused on the effects of starvation, ketogenic diet, and altered cerebral oxygen in seizures and published their first monograph entitled “*Epilepsy from the Standpoint of Physiology and Treatment*” [14]. In 1922, Cobb and Lennox published another monograph entitled “*Epilepsy and Related Disorders*” (1928) [15] and an important paper summarizing their research entitled “*The Relation of Certain Physicochemical Processes to Epileptiform Seizures*” (1929) [16]. Lennox and Cobb focused on the effects of various stimuli to the generation of epileptic convulsions such as starvation, ketogenic diet, and lack of oxygen, most of them with negative results.

During the 1940s, important discoveries were made in the field of psychomotor epilepsy. Klüver (1897–1979), a German-American psychologist, and Bucy (1904–1992), an American neuropathologist, well known for the discovery of the Klüver-Bucy syndrome, showed that changes in the behavior of monkeys could be associated with temporal lobe lesions [17]. In 1941, Jasper (1906–1999) and Kershmann proved that the temporal lobe is the site of origin of psychomotor seizures [18]. At the same period, Moruzzi (1910–1986) and Magoun (1907–1991) discovered the reticular formation in the brain [19]. Magoun continued his research with Lindsley (1907–2003) and Starzl (1926-) identifying various neural pathways within the brain and pointing out the important role in alert wakefulness as a background for sensory perception, higher intellectual activity, voluntary movements, and behaviors [20, 21]. Dawson in 1947 recorded the responses from the human scalp in response to somatosensory stimuli (somatosensory evoked potential) [22], whereas in 1949 Roberts (1920-) and Frankel discovered *γ*-aminobutyric acid (GABA) [23].

Important advances were made in the field of neuroscience and in the physiology of synapses by Eccles (1903–1997), Kandel (1929-), Spencer (1931–1977), Speckmann (1939-), Purpura, Meldrum, and others [24–38].

James Kiffin Penry (1929–1996), in 1969, published important treatises such as the series* Basic Mechanisms of the Epilepsies* and afterwards* Antiepileptic Drugs, Neurosurgical Management of the Epilepsies, Complex Partial Seizures, and their Treatment*, and* Antiepileptic Drugs Mechanisms of Action and Advances in Epileptology*. In the same year Gastaut managed to organize a meeting in Marseilles attended by 120 members of ILAE and a preliminary classification of epilepsies was presented to a commission on terminology of epilepsy. The General Assembly of the ILAE accepted the first publication of clinical and electroencephalographic classification of epileptic seizures [39, 40].

Dreifuss (1926–1997) worked on video-monitoring of absence seizures and helped in the classification of various epileptic conditions [41]. Prince et al. made the first studies of cellular phenomena of epileptic events in the human cortex [42–44]. Meldrum et al. proved that the assumption connecting brain damage from seizures as a result of hypoxia is wrong [45–47]; he demonstrated that the excessive excitatory activity is responsible for the brain cellular loss.

During the last two decades, various changes regarding the epileptic brain damage were also studied, such as the mossy fiber sprouting and synaptic reorganization [48–51].

### 2.2. The Electrophysiology of Epilepsy and the EEG

The first reference regarding the association of electric stimuli and brain activity came from the work of Fritsch (1838–1927) and Hitzig (1938–1907), who managed to cause convulsion to dogs by applying electric stimuli on the animals' cortex. Five years later, in 1875, Caton (1842–1926) examined the electrical activity of nerve-muscle preparations and explored the possibility whether similar changes in electrical potential occurred in the brain [52]. A few years later, in 1890, Beck from Cracau in the pages of Zentrallblatt for Physiologie argued the case for the priority of the electrical activity of the brain, after electrical stimulation in the brain of dogs and rabbits [53]. In 1912, Kaufman (1877–1951), a Russian physiologist, noticed the electric changes in the brain during experimentally induced seizures, associating epileptic attacks with abnormal electric discharges [54] (EEG). In the same year, Pravdich-Neminsky (1879–1952), a Ukrainian physiologist, published the first animal EEG and the evoked potential of the mammalian (dog) [55].

Two years later, Cybulski (1854–1919), a Polish physiologist and pioneer in electroencephalography, in cooperation with Jelenska-Macieszyna [56], published the first photographs of electroencephalography recording action potentials at a dog with focal epilepsy.

Important discoveries in the fields of electroencephalography were made during the 1920s and 1930s. In 1929, Berger (1873–1941), a German neurologist, reported his findings on human brain waves [57], five years after his initial recording of the first human electroencephalogram. His results brought controversy and scepticism within the scientific community, but he was neither rejected nor ignored; his results were confirmed later by Adrian (1899–1977) and Matthews [58]. In 1932, Berger reported sequential postictal EEG changes after a generalized tonicoclonic seizure, and in 1933 he published the first example of interictal changes and a minor epileptic seizure with 3/s rhythmic waves in the EEG [59, 60]. In the next few years until 1939, Berger made important observations on patients and on healthy subjects. His work on epileptic EEG was completed by Frederic Andrews Gibbs (1903–1992), an American neurologist, and Erna Leonhardt-Gibbs (1904–1987), technician and wife of Frederic, who, in collaboration with Lennox, established the correlation between EEG findings and epileptic convulsions [61–63]; Lennox and Gibbs published in 1941 their monumental monograph “*Atlas of Electroencephalography*,” in which they included also mechanical and mathematical analysis of electroencephalograms [64].

**Figure 2:**
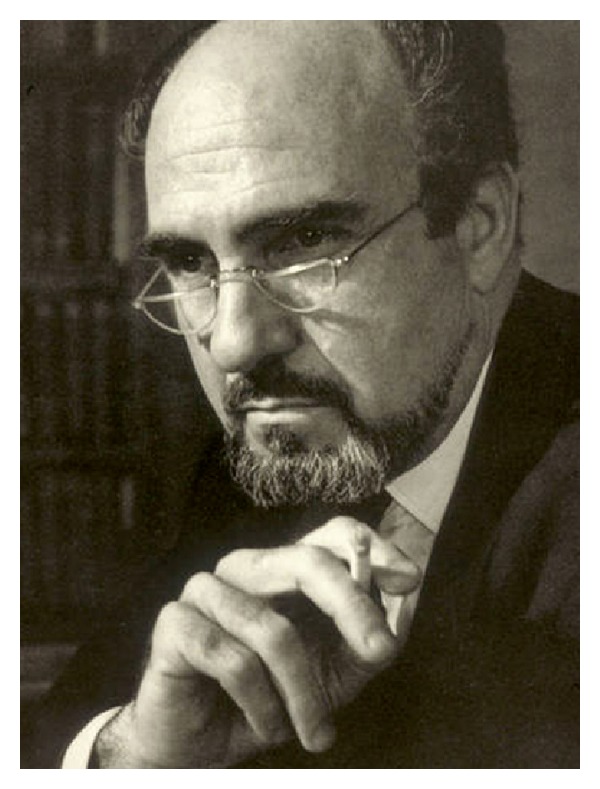
Henri Jean Pascal Gastaut (1915–1995) (adopted by public domain at http://www.lennox-gastaut.de/Krankheitsbild.112.0.html).

**Figure 3:**
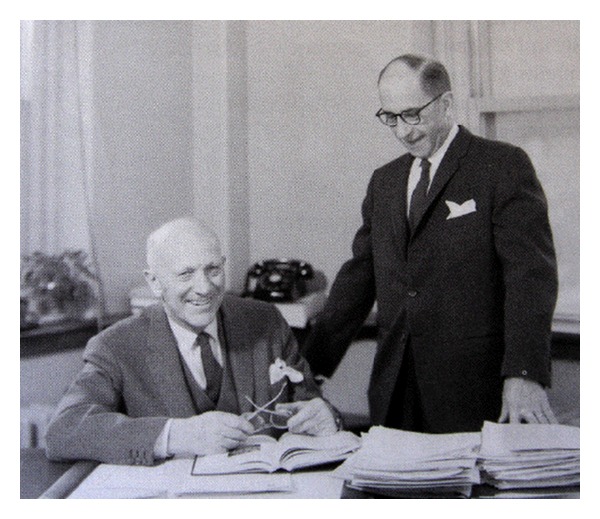
Wilder Penfield (1891–1976) on the right and Herbert Jasper (1906–1999) on the left (adopted by public domain at http://baillement.com/lettres/penfield.html).

An important and influential figure in the field of EEG whose work was intensified during the 1950s was Henri Jean Pascal Gastaut (1915–1995) (Figure 2). After his graduation in 1945 from the University of Marseilles, Gastaut worked at the laboratory of William Grey Walter (1910–1977) in Bristol learning the basics of EEG and discovered photic stimulation as an EEG seizure activator. In 1949, he went to the Montreal Neurological Institute (MNI) with Wilder Penfield (1891–1976), famous Canadian neurosurgeon, and Herbert Jasper (Figure 3) (1906–1999), a Canadian psychologist, physiologist, anatomist, chemist, and neurologist who established in 1939 an EEG laboratory and studied the role of thalamic reticular structures in the genesis of metrazol-induced generalized paroxysmal EEG discharges and developed the concept of centrencephalic seizures [65]. After his return to Marseilles, Gastaut founded the International EEG Federation and, in 1953, became the Head of the Marseilles Hospital Neurobiological Laboratories establishing a school of neurology that dominated for the next decades. In 1958, he participated in the foundation of the Toul Ar C'hoat Center in Brittany for the education of epileptic children, whereas two years later he created the Saint Paul Center for epileptic children and, in 1961, the INSERM Neurobiology Research Unit. His contribution in the study of epileptology was monumental; with his wife, Yvette, he defined five major human EEG patterns (lambda waves, pi rhythm, mu rhythm, rolandic spikes, and posterior theta rhythm) [66, 67]. He also described two syndromes under his name: Gastaut syndrome, a type of photosensitive epilepsy [68], and the Lennox-Gastaut syndrome (severe childhood encephalopathy) with onset in childhood with myoclonic seizures at night, head nodding, and drop attacks particularly prominent [69, 70]. He also studied photic and other self-induced seizures, startle epilepsy, HHE syndrome, and benign partial epilepsy of childhood with occipital spike-waves [68, 71–75].

During the 1960s, important EEG studies were conducted in animals mainly by Prince and his research team demonstrating the spikes and waves associated with synchronous paroxysmal depolarizing bursts occurring in cortical neurons [76–79] and the spike-wave complex [80]. In 1968, Falconer recognized the importance of hippocampal sclerosis in temporal lobe epilepsy [81].

### 2.3. The Patch-Clamp Technique

An important development in the field of neuroscience was that of Neher (1944-), who invented the patch-clamp method to measure the flow of current through single-ion channels [82]. Neher and Sakmann developed the patch-clamp technique for which in 1992 they received the Nobel Prize [83]. Using the patch-clump technique, the various ion channels were able to be studied and, thus, the role of calcium channels was clarified in epilepsy [84].

## 3. Therapy of Epilepsy

### 3.1. The Evolution of Antiepileptic Surgery

The first surgical procedures on epileptic patients were performed during the 19th century; Heyman in 1831 was the first one to perform a surgery to an epileptic patient due to a brain abscess. Surgical excision was performed on November 25, 1884, by Dr. Godlee in the National Hospital of London. In 1880, Wilhelm Sommer (1852–1900), German neurologist and psychiatrist, described precisely Ammon's horn lesions and epileptic manifestations part with sensible occurrence. Both Theodor Kocher (1841–1917), a Swiss surgeon from Bern, Nobelist, and pioneer in epileptic surgery, and Harvey Cushing (1869–1939), father of modern neurological surgery, in Baltimore dealt with posttraumatic epileptic disorders especially with patients displaying high endocranial pressure [85, 86]. In 1886, Horsley (1875–1916) excised an epileptogenic posttraumatic cortical scar at the National Hospital of London in a 23-year-old man under general anesthesia and discussed his choice of anesthesia: “*I have not employed ether in operations on man, fearing that it would tend to cause cerebral excitement; chloroform, of course, producing on the contrary, well-marked depression*.” [87]. In Germany, Krause (1857–1937) and Foerster (1873–1941) refined Horsley's technique [88, 89].

At the beginning of the 20th century, Dandy (1886–1946) introduced hemispherectomy as a neurosurgical procedure in 1923 [90]. However it was not until the 1930s than important advances were made in epileptic surgery. The notion of operating the epileptogenic focus was introduced by Gibbs and Lennox in 1938 [91]. The introduction of EEG into epilepsy surgery was important in the development of surgical techniques.

Penfield along with Jasper and Theodore Brown Rasmussen (1910–2002) in the Neurologic Center of University of Montreal also contributed importantly to the evolution of the surgery of epilepsy [92, 93]. Penfield applied the Foerster method for removing epileptogenic lesions on an epilepsy patient. After founding the Montreal Neurological Institute (MNI), in 1934, in collaboration with Jasper, he invented the* Montreal procedure* for the surgical treatment of epilepsy. According to the Montreal procedure, through the administration of local anesthetic, the surgeon removes part of the skull to expose brain tissue and, by the use of probes, the conscious patient describes to the surgeon his/her feelings so that the surgeon can identify the exact location of seizure activity. Then the surgeon proceeds in the removal of brain tissue in this location reducing the side effects of surgery [94]. Through his operations, Penfield was able to identify various brain centers and to create maps of the sensory and motor cortices of the brain. Research in MNI focused also on other areas of epileptology such as neurochemistry, oncology, and brain angiology. Penfield perfected and established his surgical procedures as a treatment of choice in intractable epilepsy, especially of neocortical regions [94–96]. In 1954, Penfield published with Jasper one of the greatest classics in neurology,* Epilepsy and the Functional Anatomy of the Human Brain *[93].

Around the same period, van Wagenen and Herren (1897–1961), Chief of Neurosurgery at the University of Rochester Medical Center (URMC), performed and perfected the procedure of callosotomy [97]. Bailey (1892–1973), an American neuropathologist, neurosurgeon, and psychiatrist, known for his work on brain oncology, was the first to attempt temporal lobectomies for psychomotor seizures and the first to use electrocorticography for intraoperative localization [98]. One should also mention the method of hemispherectomy introduced by McKenzie (1892–1964) [99] and Krynauw in 1950 [100].

Bailey and Gibbs in 1951 employed the EEG as a guide to perform temporal lobe surgery [98], whereas, in 1953, Falconer, a neurosurgeon from New Zealand, in London, introduced the en bloc anterior temporal lobe resection and the term mesial temporal sclerosis [101]. The work of Margerison and Corsellis led to the term of hippocampal sclerosis [102], a pathological entity which was initially described almost 80 years earlier by Sommer in 1880 [103]. Niemeyer, in 1958, suggested a more selective procedure of resection of the mesiobasal limbic structure [104], a technique which was later on abandoned.

The next important step in the field of antiepileptic surgery was done by Tailarach and his team. In 1957, Tailarach (1911–2007) published his stereotactic atlas, a work that changed the future of epilepsy neurosurgery the next decade [105]; Marcel David adopting Tailarach's views supported the creation of an operating room in which stereotactic surgery would take place. Within this operating room teleradiography would take place and the use of parallel X-ray beams would avoid distortions of skull, vessels, ventricles, and the frame and grids used for guiding the placement of intracranial electrodes. The first stereotactic surgery operating room was opened in Sainte-Anne in 1959 [106]. Tailarach's team obtained two members, Alain Bonis and Gabor Szikla. In 1962, the term stereoelectroencephalography (SEEG) was introduced by Talairach and Jean Bancaud (1921–1993). Their method brought a revolution in the surgery of epilepsy, since it allowed investigative presurgical and therapeutic surgical phases to be completely dissociated. Tailarach and Bancaud employing their technique showed that lesional and irritative zones had a variable topographic relationship within the epileptogenic zone [14]. Tailarach's method allowed the individualization of epileptic surgery for each patient [18, 19].

During the 1960s, Bogen and Vogel reintroduced the procedure of callosotomy [107] as a procedure for certain cases of pharmacoresistant epilepsy with severe atonic akinetic seizures. In 1961, White published a comprehensive review on the surgical procedure of hemispherectomy summarizing the results of 269 published cases [108] in the treatment of infantile-type hemiplegia and seizures. In 1969, Morell and Hanbrey introduced “multiple subpial transection” (MST) for nonresectable epileptic foci [108].

At the beginning of the 1980s, in the field of antiepileptic surgery, MTLE suggested selective amygdalohippocampectomy (AHE) with the trans-Sylvian approach, replacing the anterior temporal lobe resection [109]. The advent of modern diagnostic techniques such as MRI, PET, and SPECT (single photon emission tomography), ^31^P and ^1^H-MR spectroscopy, and MEG (magnetoencephalography) revolutionized epileptic surgery, as well. The application of microsurgery led to selective operations with less complications; such procedures include “selective amygdalohippocampectomy” [109], the innovation of older ones, anterior callosotomy, subtotal functional hemispherectomy, and extended multilobar resections, and the introduction of new operative techniques such as multiple subpial transection [109] and gamma knife.

### 3.2. Drug Therapy

As far as therapies and the neurophysiology of epilepsy are concerned, much were already known during the second half of 19th century. Treatment of epilepsy till that time mostly consisted of herbal and chemical substances. In 1857, Sir Locock (1799–1875) discovered the anticonvulsant and sedative traits of potassium bromide and began treating his patients. From that point, potassium bromide became a choice treatment for humans with epileptic seizures and nervous disorders until the 1912 discovery of phenobarbital [110].

In 1912, Hauptmann (1881–1948), a German physician, introduced phenobarbital in the therapy of epilepsy, one of the first antiepileptic drugs [111]. Phenobarbital was brought to market by the drug company Bayer using the brand Luminal. Hauptmann administered Luminal to his epilepsy patients as a tranquilizer and discovered that their epileptic attacks were susceptible to the drug. The introduction of animal models in the study of the anticonvulsant properties of various substances will contribute to the development of new antiepileptic drugs.

The next drug introduced in the therapy of epilepsy was phenytoin in 1938. Although phenytoin was already known from 1908 and was synthesized by Heinrich Biltz (1865–1943), there was no interest for that drug since it did not have any sedative properties. Merritt (1902–1979), an eminent academic neurologist, along with Putnam (1894–1975), discovered, in 1938, the anticonvulsant properties of phenytoin (Dilantin) and its effect on the control of epileptic seizures publishing their results in a series of papers [112–115]. Phenytoin became the first-line medication for the prevention of partial and tonic-clonic seizures and for acute cases of epilepsies or status epilepticus, giving an alternative therapeutic choice for patients not responding to bromides or barbiturates. In 1946, a new antiepileptic drug was added in the quiver of antiepileptic therapy, trimethadione; it was reported by Richards and Everett to prevent pentylenetetrazol-induced seizures and to be effective especially in absence seizures [116].

During the 1950s, new drugs came up such as carbamazepine in 1953 [117], primidone in 1954, ethosuximide in 1958 by Vossen [118], sodium valproate in 1963 by Meunier et al. [119], and sultiame. Buchtal and Svensmark were the first ones in 1960 to measure the levels of the antiepileptic drugs in the blood [120]. Although carbamazepine and valproate were available in Europe during the 1960s, no other drug was licensed in the USA. The development of carbamazepine was based on the neuroleptic drug chlorpromazine from Firma Rhône-Poulenc in Lyon. Jean Pierre (1907–1987) and Pierre Deniker (1917–1998), French psychiatrists, used chlorpromazine in Centre Hospitalier Sainte Anne in Paris to treat patients with schizophrenia. However, research on neuroleptic drugs continued in Geigy labs; carbamazepine was synthesized by Schindler and Blattner (1921-?) at J. R. Geigy AG, Basel, Switzerland, 1953, in the course of development of another antidepressant drug imipramine [117]. Initial animal screening showed that carbamazepine was effective against trigeminal neuralgia, which was confirmed by clinical trials [121]. Antiepileptic effects were reported in 1963 and 1964 [122, 123]. It was used as an anticonvulsant drug in the UK since 1965 and has been approved in the USA since 1974. The reason for the delay of approval in the USA was due to reports of aplastic anemia caused by the drug [124]. Ethosuximide was first introduced in clinical practice in the early 1950s for the therapy of absence “petit mal.”

In 1967, valproate came up as a new promising antiepileptic drug. Valproate was initially synthesized in 1881 by Beverly Burton in the USA and was initially employed as an organic solvent [125]; his research on valproate begun in Würzburg, Germany. The anticonvulsant properties of valproate were reported by Pierre Eymard, who worked at Firma Berthier laboratories in Grenoble, and it was first released as antiepileptic drug in France in 1967 [126] after the publication of preclinical studies by Garraz et al. in 1964. During 1970, it received license to other European countries, but in the USA it was not licensed before 1978.

In 1970, Penry and Cereghino were employed in designing clinical trials for antiepileptic drugs (AEDs). Harvey Kupferberg joined their team and together they developed a methodology for measuring the blood-levels of albutoin, an experimental drug which was proved to be ineffective in epilepsy. The first edition of* Antiepileptic Drugs *came forth as a result of their research efforts in 1972 [127]. Carbamazepine was the first drug to be licensed by the FDA based on the results of clinical trials. Pippenger (1939-) developed methods for measuring blood-levels of AEDs [128]. Other antiepileptic drugs introduced during the 1970s were clobazam (1,5-benzodiazepine) (1970), clonazepam (1,4-benzodiazepine) (1970), and piracetam.

The last decade newer antiepileptic drugs such as vigabatrin (1989), lamotrigine (1990), oxcarbazepine (1990), gabapentin (1993), felbamate (1993), topiramate (1995), tiagabine (1998), zonisamide (1989 in Japan and 2000 in the USA), levetiracetam (2000), stiripentol (2002), pregabalin (2004), rufinamide (2004), lacosamide (2008), eslicarbazepine (2009), and perampanel (2012) were used. FDA ended clinical use of felbamate in 1994 due to its association with complications. The newer generation antiepileptic drugs including vigabatrin, felbamate, gabapentin, lamotrigine, tiagabine, topiramate, levetiracetam, oxcarbazepine, zonisamide, pregabalin, rufinamide, and lacosamide have improved tolerability and safety compared to their older counterparts. Stiripentol, pregabalin, rufinamide, lacosamide, eslicarbazepine, and perampanel are licensed for adjunctive use only. The research in antiepileptic drugs is an active field and many drugs are currently under development in clinical trials including eslicarbazepine acetate, brivaracetam, and retigabine.

In phase III clinical trials (which used eslicarbazepine doses of 400, 800, and 1200 mg/day), eslicarbazepine was well tolerated, with the most common AEs reported to include dizziness, headache, and somnolence [129–132]. Two large-scale, phase III clinical trials have been conducted for retigabine. In both studies, AEs leading to discontinuation included dizziness, somnolence, headache, and fatigue [133–136].

### 3.3. The Idea of Ketogenic Diet

Ketogenic diet was used for the first time in the treatment of epilepsy in 1911 by the French physicians Guelpa and Marie. It was introduced as a diet full of fats and low in proteins and carbohydrates and managed to treat 20 children and adults with epilepsy reporting decrease in the number of seizures [137]. However, fasting and other diets were employed for the treatment of epilepsy since the Hippocratic era [138].

In 1922, Hugh Conklin, an osteopathic physician from Michigan, applied this diet to epileptic patients with encouraging results. Conklin believed that epilepsy was due to toxins that damage the brain and so he obliged his patients to a strict diet. In his papers, he wrote,* “I restrict from my patients any kind of food except of water for as long as their physical condition allows it.”* Conklin had a personal interest in ketogenic diet, since by this way he tried to cure his nephew, who suffered from drug-resistant epilepsy. Several papers have been published about the usefulness of ketogenic diet and the indications that will lead to the beginning of such a treatment [139, 140]. Further studies were published by Talbot [141], Helmholz [142], Lennox [143], and Bridge and Iob [144]. Charles Howland, a wealth New York lawyer, funded his brother John Howland to search whether there was a scientific basis for the success of the starvation treatment by which his epileptic son was treated [145, 146]. Dr. John Howland, Professor of paediatrics, John Hopkins Hospital, used this funding to create the first laboratory at the Harriet Lane Home for Invalid Children. John Howland Memorial Fund was established at John Hopkins Hospital and supported the research on the ketogenic diet [147]. Although multiple investigations have been performed, the anticonvulsive mechanisms of ketogenic diet remain unexplained. In the years to follow, many authors published articles with positive or negative results. Then, this therapy was forgotten for many years, since progress in pharmaceutical therapy was in the foreground. In the last 20–30 years, KD experienced a revival, especially in the Anglo-American world where it is established as a treatment option for therapy-resistant infantile epilepsy. Livingston [148], Hopkins and Lynch [149, 150], and Huttenlocher [151] were the advocates of this direction. The multitude of side effects and procedural problems however reduced the initial euphoria. Only as recently as 1996, the* Institute Charlie* (the institute was named by a father of a child with epilepsy which was treated with* KD*) educated the public about the benefit of KD, organized seminars and training courses, and published a book entitled* The Epilepsy Diet Treatment: An Introduction to the Ketogenic Diet*. More recently new versions of ketogenic diet such as the modified Atkins diet have been employed successfully for the treatment of children and adults with refractory epilepsy [152, 153] and are especially recommended in cases of pharmacologically intractable epilepsy.

### 3.4. The Technique of Vagus Nerve Stimulation

An important advance in epilepsy treatment was the development of the technique of vagus nerve stimulation (VNS), especially for patients experiencing serious adverse effects of antiepileptic drugs. VNS involves implantation of a programmable signal generator (neurocybernetic prosthesis NCP) in the chest cavity, and the stimulating electrodes carry electrical signals from the generator to the left vagus nerve [154].

### 3.5. Complimentary Treatments for Epilepsy

During the last two decades a series of alternative or complimentary therapies have emerged in the therapy of epilepsy. These include relaxation therapies such as massage, aromatherapy, reflexology and chiropractic therapy, holistic therapies such as herbal medicine (St. John's Wort, evening primrose oil), homeopathy, Ayurvedic medicine, and traditional Chinese medicine (herbal remedies plus acupuncture), traditional and psychological therapies such as autogenic training, neurofeedback, and other psychological therapies, and music therapy. Although some of those therapies seem to have an effect, most of them are considered as complementary therapies and more studies are needed in order to establish their therapeutic effect and their usefulness in everyday clinical practice [155]. Recent studies have pinpointed the use of cannabidiol and medical marijuana for the treatment of epilepsy [156].

## 4. The Evolution of Thoughts around Epilepsy: Society and Science Cooperate for the Good of Epileptic Patients

Just before the turn of the twentieth century, in 1898, William Pryor Letchworth (1823–1910), a businessman devoted to charities, and Frederick Peterson (1859–1938), an eminent American neurologist and Professor of neurology, Columbia University, organized the first National Association for the Study of Epilepsy and the Care and Treatment of Epileptics in the USA and the Craig Colony in Sonyea, the first comprehensive public epilepsy center, along with other eminent physicians such as Roswell Park (1852–1914), Professor of surgery, University of Buffalo Medical School, and a surgeon, Buffalo General Hospital, William P. Spratling (1863–1915), American neurologist, known for his studies in epilepsy, and Frederick Munson [157, 158]. William Spartling introduced for the first time the term “epileptologist” for a physician specializing in epilepsy.

During World War I, Pearce Bailey organised the systematic examination of military recruits creating a database with epidemiological data on epilepsy. The same year is considered to be the founding year of the American Epilepsy Society (AES) in commemoration of a joint meeting focusing on epilepsy organised by the Association for the Research in Nervous and Mental Disease (ARNMD) and the American League Against Epilepsy (ALAE). The proceedings of this meeting recapitulated the state of the art on epilepsy research and stimulated further research [159]. The first president of AES was Dr. Charles Dair Aring (1904–1998), a renowned American neurologist and pioneer in medical education in the USA.

The beginning of the 1950s was marked by the establishment by the US congress and the American Academy of Neurology of the National Institute of Neurological Diseases and Blindness (NINDB), now known as the National Institute of Neurological Disorders and Stroke. The main purpose of this institute was to study and treat the neurological and psychiatric casualties of World War II under the direction of Bailey. Bailey recruited in his team Maitland Baldwin (1918–1970), a famous American neurosurgeon, as the Chief of surgical neurology, Bethesda, Donald Bayley Tower (1919–2007), in 1953, as the Chief of the section on clinical neurochemistry, and Cosimo Ajmone-Marsan (1918–2004), as the Head of the electroencephalography branch in 1954. As a result of their cooperation, a treatise on temporal lobe epilepsy was published [160].

During the 1960s, in 1961, the International Bureau for Epilepsy (IBE) was established as an organisation of laypersons and professionals interested in the medical and nonmedical aspects of epilepsy (http://www.ibe-epilepsy.org). In 1962, the US Public Health Service Surgeon General created the Neurological and Sensory Disease Control Program (NSDCP) supporting research on epilepsy in various ways. In 1965, Anthony Joseph Celebrezze (1941–2003), Secretary of the Department of Health, Education, and Welfare, organized a meeting for the expansion of epilepsy research and services. As a result of it, in 1966, Surgeon General William Stewart (1921–2008) created the Surgeon General's Public Health Service Advisory Committee on the Epilepsies, whereas, in 1969, the Society for Neuroscience was established. Stewart created two subcommittees: one with David Daily entitled* Service Training Subcommittee *with Terrance Capistrant and James Cereghino as Executive Secretaries and a second one with Arthur Ward entitled* Research Training Subcommittee *with William Caveness and J. Kiffin Penry as Executive Secretaries [65].

In 1970, a classification of epilepsies was proposed by the International League Against Epilepsy [161]. This classification was revised several times through the last decades, in order to clarify terms and meanings and to avoid any confusion towards the understanding of epilepsy. In 1975, the US Commission for the Control of Epilepsy and its Consequences was established under the direction of Dr. Richard H. Masland and Dr. David Daly, creating a plan for nation-wide action on epilepsy.

During the last two decades, epileptics are being evaluated psychologically and socially and, before 1990, quality of life tools were developed. In 1981, the International League Against Epilepsy (ILAE) published the first classification of epilepsies which were discussed extensively and revised in 1989 [162–164]; the classification and terminology for epileptic seizures and syndromes provided a fundamental instrument for diagnosing, organizing, and differentiating the epilepsies.

During the 1990s, the decade of the brain, WHO in collaboration with ILAE and IBE launched the Global Campaign Against Epilepsy in 1997, in order to bring epilepsy out of the shadows and to further improve diagnosis, treatment, prevention and social acceptability. In 1993, the ILAE defined fever seizures as the seizures occurring in childhood after the age of 1 month, usually between 3 months and 6 years old, associated with a febrile illness, not caused by an infection of central nervous system (CNS), without previous neonatal or unprovoked seizure, and not meeting criteria for other acute symptomatic seizures [165]. In 2001, the ILAE Task Force on Classification and Terminology under Engel proposed a diagnostic scheme for people with epileptic seizures and with epilepsy [166] which was also supported in 2006 by the ILAE Classification Core Group [167]. ILAE's classification of epilepsies and convulsive disorders was extensively discussed and new classification was suggested but not implemented since 2010 when ILAE published a new classification [168, 169].

## 5. The Contribution of Imaging Techniques in the Diagnosis of Epilepsy

After the end of World War I, Dandy (1886–1946), an American neurosurgeon, described in 1918 and 1919, pneumoventriculography and pneumoencephalography [170–172], the first imaging techniques of the brain with the use of X-rays. For his discovery, he was nominated by Hans Christian Jacobaeus for the Nobel Prize in 1933. In the field of imaging, it was not until 1972 when computerized tomography (CT) was invented by the British engineer Godfrey Hounsfield (1919–2004), EMI Laboratories, UK, and by the South African physicist Allan MacLeod Cormack (1924–1998), Tufts University, Massachusetts [173]. The use of ^18^F-fluorodeoxyglucose for determination of local cerebral localization began in 1977 [174, 175].

During the last decades great steps have been taken to the diagnostic approach of epilepsy by the use of neuroimaging. Techniques as magnetic resonance tomography (MRI), SPECT, and PET contributed to the diagnosis of pathological areas of the brain, such as tumors, cortical/subcortical dysgeneses, inflammation, strokes, vascular dysplasia, and posttraumatic insult. Newer imaging techniques in the diagnosis of epilepsy include functional MRI [176] (fMRI), clinical proton MR spectroscopy [177], and magnetoencephalography (MEG) [178].

## 6. Genetics in Epilepsy

The first connection between heredity and epilepsy was made in 1903 by Lundborg (1868–1943), a Swedish physician, notorious for his views on eugenics and racial hygiene, who published his research on the genetics of progressive myoclonic epilepsy first described by Heinrich Unverricht in 1891 (1853–1912) [179]; his analysis was pioneering since he was able to trace back the disease in the family since the 18th century. In that way, Lundborg was also a pioneer in the study of human genetics.

The concept of eugenics became an issue in the control of epilepsy during the 1930s; in 1936, the American Neurological Association Committee for the Investigation of Eugenical Sterilization published a report [180] stating that sterilization of epileptics should be voluntary and conducted under supervision and only with patient consent.

The most important evolution, however, in the field of the genetics of epilepsy, took place during the last twenty years; in 1989, Leppert was the first to identify the link between chromosome 20 and idiopathic human epilepsy syndrome in a family with benign familial neonatal convulsions [181]. Epilepsy still remains a field of active research, occupying different medical specialties. The growing evidence on the connection between various genes and epilepsies is the cutting edge of modern epilepsy research, and in the next decades new exciting discoveries are going to change epileptology [182]. Recently, in 2011, Engel published the identification of reliable biomarkers which would greatly facilitate differential diagnosis, eliminate the current trial-and-error approach to pharmacotherapy, facilitate presurgical evaluation, and greatly improve the cost-effectiveness of drug discovery and clinical trials of agents designed to treat, prevent, and cure epilepsy [167].

## 7. Conclusions

The fascinating history of epilepsy is connected with the history of humanity; early reports on epilepsy go back to the ancient Assyrian and Babylonian texts, scanning a period of almost 4,000 years. The first hallmark in the history of epilepsy is the Hippocratic texts which set in doubt the divine origin of the disease. Major advances in the understanding of epilepsy came much later, during the 18th and 19th century; theories on epilepsy during this period are formulated on a solid scientific basis and epileptics are for the first time treated as patients and not as lunatics or possessed. During this period, experimental studies were conducted and advances made in the pathology of the disease and the connection of epilepsy with various psychiatric symptoms. The work of John Hughlings Jackson was preceded by a plethora of studies by Dutch, German, English, and French physicians who evolved scientific thought and performed thorough studies on epilepsy. The advent of the 20th century led to the in-depth understanding of the mechanisms of the disease, the development of effective drugs, and neuroimaging methods. Last but not least, one should mention the important advances in the molecular biology of the disease and the connection of various genes with various forms of epilepsy.
